# Stakeholder perceptions of mental health stigma and poverty in Uganda

**DOI:** 10.1186/1472-698X-9-5

**Published:** 2009-03-31

**Authors:** Joshua Ssebunnya, Fred Kigozi, Crick Lund, Dorothy Kizza, Elialilia Okello

**Affiliations:** 1 Department of Mental Health and Community Psychology, Makerere University, Makerere, Uganda; 2 Butabika National Referral and Teaching Mental Hospital, Butabika, Uganda; 3 Department of Psychiatry and Mental Health, University of Cape Town, Cape Town, South Africa; 4 Department of Psychiatry, Faculty of Medicine, Makerere University, Makerere, Uganda

## Abstract

**Background:**

World wide, there is plentiful evidence regarding the role of stigma in mental illness, as well as the association between poverty and mental illness. The experiences of stigma catalyzed by poverty revolve around experiences of devaluation, exclusion, and disadvantage. Although the relationship between poverty, stigma and mental illness has been documented in high income countries, little has been written on this relationship in low and middle income countries.

The paper describes the opinions of a range of mental health stakeholders regarding poverty, stigma, mental illness and their relationship in the Ugandan context, as part of a wider study, aimed at exploring policy interventions required to address the vicious cycle of mental ill-health and poverty.

**Methods:**

Semi-structured interviews and focus group discussions (FGDs) were conducted with purposefully selected mental health stakeholders from various sectors. The interviews and FGDs were audio-recorded, and transcriptions were coded on the basis of a pre-determined coding frame. Thematic analysis of the data was conducted using NVivo7, adopting a framework analysis approach.

**Results:**

Most participants identified a reciprocal relationship between poverty and mental illness. The stigma attached to mental illness was perceived as a common phenomenon, mostly associated with local belief systems regarding the causes of mental illness. Stigma associated with both poverty and mental illness serves to reinforce the vicious cycle of poverty and mental ill-health. Most participants emphasized a relationship between poverty and internalized stigma among people with mental illness in Uganda.

**Conclusion:**

According to a range of mental health stakeholders in Uganda, there is a strong interrelationship between poverty, stigma and mental illness. These findings re-affirm the need to recognize material resources as a central element in the fight against stigma of mental illness, and the importance of stigma reduction programmes in protecting the mentally ill from social isolation, particularly in conditions of poverty.

## Background

Stigmatization of individuals diagnosed as having serious mental illness has been observed across the world [1]. Stigma does not stop at the illness: it marks those who are ill and their families over generations, institutions that provide treatment, psychotropic drugs and mental health workers [2]. The family members who help care for people with mental illness report feeling stigmatized as a result of their association with the loved one having mental illness [3]. The presence of stigma starts a vicious cycle that leads to discrimination in all walks of life [1], decreasing self esteem and self confidence, and adversely affecting social engagement. The resultant reduction in self-esteem may increase disability by reducing access to social and financial resources [4].

While there is considerable descriptive evidence regarding the role of stigma in mental illness, there is also a large body of evidence from high, middle and low-income countries, demonstrating an association between poverty and mental illness [5,6]. Poverty is not only a consequence of mental ill-health but also often precedes mental illnesses such as depression and anxiety; making it an important risk factor for mental illness and other negative outcomes [5-7].

For mental health consumers, poverty may mediate the impacts of mental illness [8] although more work is needed to understand this relationship [9]. The social relationships made difficult by reduced interpersonal functioning may be further strained by poverty. Lack of income has been said to negatively affect opportunities for social network development and social integration [10], and to work against the empowerment of mental health service consumers [11]. Poverty therefore has grave implications for people's health, education, social relations and social inclusion [12].

While mental illness affects individuals at all economic levels, many people with mental health problems end up in poverty due to the stigma attached to being labeled mentally ill. This stigma frequently makes it difficult for them to enter or re-enter the workforce [13]. If employed, individuals with mental illness may suffer disparaging remarks at work due to a lack of sympathy and understanding [14]. The physically demanding nature of unskilled labour (a hallmark of most African economies) also makes it difficult for disabled people to be involved in labour intensive activities. This situation is made worse by outright social exclusion of disabled people that constrains disabled people's participation in the job market [15]. The experiences of stigma catalyzed by poverty revolve around experiences of devaluation, exclusion, and disadvantage [16], which may be internalized in the forms of self-hatred, self-isolation, and shame [17].

Thus three powerful forces: material deprivation, the stigma of mental illness and the stigma of poverty interact in the lives of people living with mental illness in conditions of poverty. Although the relationship between poverty, stigma and mental illness has been documented in high income countries, little has been written on this relationship in low and middle income countries; and to our knowledge, this has not been researched in Uganda. This is an important issue given the levels of extreme poverty and social isolation endured by people with severe mental illness in low and middle-income countries. Within East Africa, Uganda has the highest level of income poverty, with an annual income per capita of $300 as compared to $350 and $580 for Tanzania and Kenya respectively [18]. Although there is little data on the prevalence of mental illness in Uganda, one third of the Ugandan population has been said to have some form of mental disorder [19]. The 2006 household survey by the Uganda Bureau of Statistics indicated that 4% of the households in Uganda had at least one member with a mental disability [20]. People with mental illness are not only among the poorest in society, but they remain poor for very long periods of time, and from generation to generation [15].

In this paper, we set out to explore the opinions of a range of mental health stakeholders in Uganda regarding the role of poverty and stigma in the experience of people living with mental illness. Specifically we set out to assess the way in which these stakeholders constructed notions of poverty, stigma, mental illness and their relationship in the Ugandan context. This paper forms part of a wider study that aims to explore the policy interventions required to address the vicious cycle of mental ill-health and poverty in Ghana, South Africa, Uganda and Zambia [21].

## Methods

Semi-structured interviews (SSIs) and focus group discussions (FGDs) were conducted with a variety of mental health stakeholders in Uganda. Individual SSIs were used as they are an effective qualitative method for learning about the perspectives of individuals related to a particular topic [22]. These interviews also allowed for the detailed exploration of a particular individual's point of view. FGDs were conducted with some relatively homogenous groups of participants (such as nurses or teachers) in order to elicit participants' views; to document the discussion and interactions between these participants in relation to particular topics; and to capture a range of opinions within these groups, using the limited time and resources that were available.

Selection of the participants was done purposefully, based on a number of principles: participants represented a range of key mental health organizations in Uganda; they held specialized knowledge or had specific experience related to mental health policy, services and poverty; or they represented a variety of perspectives on mental health, poverty and stigma. In total, 62 semi-structured interviews and 6 focus group discussions (each consisting of 5–9 participants) were conducted over a 6-month period.

The participants who were interviewed included stakeholders from various sectors as follows:

1. Health Sector (6 Policy makers, 4 Programme managers, 3 Facility managers and 4 health service providers)

2. Education Sector (3 Senior Education Officers and managers, 1 Inspector of schools)

3. Law and justice sector (2 Magistrates and 2 Police chiefs)

4. Gender and Social Welfare department (1 commissioner)

5. Legislators/Politicians (4 Parliamentarians and 1 Minister)

6. Media (3 from print media and 3 from electronic media)

7. Non-Government Organizations in mental health (3)

8. User Support Organizations (1)

9. Academic and Research institutions (4)

10. Housing department (1)

11. Professional Associations (1)

12. External Development Partners/donor agencies (4)

13. Private Sector (1)

14. Religious Leader (1)

15. Traditional Healers (2)

16. Mental Health Service users (7)

The 6 Focus Group Discussions (FGDs) were conducted with homogenous groups, consisting of people of the same background as follows:

1. Mental health nurses, representing 7 health sub-districts in an urban district (7 participants).

2. A mix of general nurses and mental health nurses at the National Mental Hospital (9 participants).

3. Secondary school teachers from 5 schools in an urban district (7 participants).

4. Secondary school teachers from 5 schools in a rural district (8 participants).

5. Primary school teachers from 4 schools in a rural urban district (5 participants).

6. General nurses in a rural district (8 participants)

Of the 106 participants, 48 (45%) were female. The majority of the female participants were from the health and education sectors, specifically nurses and teachers respectively. These two were also the most represented sectors in the study. All the participants were adults aged 19 – 72 years of age; with the majority being in their thirties and forties. All 7 mental health service users were receiving services at the National Mental Hospital, and were mentally well at the time of the study. Four were males and three were females. Of the four males, one was a well known man in his early forties, who had just lost his political career after having suffered with a mental breakdown. Another one was a health worker, and one of the founder members of the national user support organization. Another one was a young man in his late twenties, and a University drop-out. The last one was an unemployed man who had battled with mental illness for nearly 20 years. Of the 3 female users, one was a prominent business person. The second one was a 19-year-old woman who had dropped out of high school due to her mental illness. The last one was a young woman in her mid twenties and a prominent member of a regional user support group, who had lost her job as secretary in some organization following a manic episode. Family members of mental health service users were not interviewed as a separate group of stakeholders. However, several of the other stakeholders interviewed were also family members of mental health service users and they brought this experience to their interviews.

With the exception of two interviews, the interviews and focus group discussions were conducted in English. The two users who could not speak English freely were interviewed in the local language, and the interviews were translated to English. Written informed consent was obtained from all the participants. Ethics approval was provided by the Ethics committee and the office of the Director General of Health Services in the Ministry of Health, Uganda. The interviews and focus group discussions were audio-recorded and transcribed verbatim. The transcriptions were then coded and entered into NVivo7 qualitative data analysis software.

Thematic analysis of the data was conducted using a framework analysis approach. This approach was explicitly developed in the context of applied research, and is gaining popularity in health policy and systems research [22]. By this approach, certain themes and sub-themes were collectively agreed upon by the investigators at all the research sites, based on the objectives of the study. A single framework for analysis was thus developed, and the transcriptions were coded on the basis of this pre-determined coding frame. Thereafter specific themes emerging from the interviews were added into the framework in the process of conducting the analysis, and transcripts were coded accordingly. All the four investigators at the Uganda site participated in the coding and analysis of the data. Coding was initially done on paper for the printed transcripts of the interviews. Two of the authors initially did the coding on paper, before data were entered and coded into Nvivo by a third author. The coded material was then checked by partners at Leeds University for consistency.

## Results

### Poverty and mental illness

Most participants identified poverty as a major risk factor for mental illness. Poverty was reported to be an important cause of distress that might result in significant mental health problems. According to participants, many poor and unemployed people, especially the uneducated, attempt to cope with their frustrations and social problems by resorting to alcohol and other illicit drugs, which make them more susceptible to mental health problems. This was affirmed by some participants' research experience:

"...*What we found out in our baseline survey was that people feel they have become mentally ill because of poverty. They are poor, they are restless, always worried, they don't sleep, they do abc..." *(SSI, mental health professional, NGO).

Some of the participants described the relationship as a vicious cycle, and maintained that while poverty is a contributory factor for mental illness, poverty can also be a result of mental illness. Service users noted that in addition to people with mental illness being unproductive during the time they are hospitalized or on treatment, carers also spend much time nursing the sick relatives. This subsequently lowers their productivity, resulting in significant economic decline. One user specifically described the recurrent nature of mental illness as characterized by high expenses and no productive work, often leading to financial loss:

"...*when I relapse, I have to use all the money and go back to zero. So, I have to begin afresh whenever I recover" *(SSI, mental health service user)

It was further reported that people with mental illness sometimes become destructive, leading to strained relationships with family members and neighbours, and a need to spend money on the resolution of disputes at local courts. This further encroaches on their meagre resources. A few participants however did not refute the relationship, but also believed that the wealthy are equally prone to mental health problems just as the poor. One mental health professional was quoted as saying:

"...*When you have a population and you look at the mental state of the population according to the social class, the distributions are U...I mean when you plot, you get a normal distribution curve. Those who are the poorest have a higher rate of mental disorders, and those who are the richest also have a higher rate of mental disorders*" (SSI, Mental Health professional and Researcher)

### Mental health-related stigma and service delivery

Mental illness continues to be regarded as a unique illness that is highly stigmatized by both high and low income groups. The stigma attached to mental illness was found to be mostly associated with belief systems regarding the causes of mental illness. According to some respondents, members of the public often consider mentally ill people to be possessed by evil spirits or paying a price for their bad deeds. The entrenched nature of stigma against mental illness was reflected in the responses by some of the presumably well informed participants who used demeaning language while referring to the mental hospital and the patients. One participant said:

"...*because when you visit Butabika [psychiatric hospital] during working hours, you will think Butabika is a place for normal people. It is cleaner than many secondary schools...and even many other hospitals. You can think the people there are normal*" (SSI, Member of Parliament)

In terms of service delivery, the majority of the participants believed that stigma against mental illness played a major role in the inequitable allocation of resources. Many respondents stated that with the inadequate resources available, mental health was usually allocated a very small proportion of the health budget, disproportionate to the disease burden. This was attributed to the misperception and high levels of stigma attached to mental health. In some general health facilities, the managers reported finding a great deal of resistance from facility administrators who are non-health professionals when it comes to allocation of resources and medical supplies to mental health. Facility administrators tended to view the mentally ill patients as 'gone cases', onto whom resources should not be 'wasted' because they are not expected to recover and become productive again:

"...*because whenever we are buying drugs, again they say but for who? You know so it becomes a bit of a problem... even now in the administration, apart from us who are medical, the other people who are non medical look at those people as mad... you know...they think it is wastage of money to buy them drugs. So it becomes a bit of big problem*" (SSI, Hospital Medical Superintendent, urban district).

Users and mental health advocates noted that stigmatization can be more destructive and disabling than the illness itself, and is a major obstacle to help seeking.

*"...So, the disability comes in here.... it is the social disability which disables people. It comes because of the stigma. They are highly stigmatized....once they are labeled and even...sorry to comment on psychiatrists but when you are in hospital for example, instead of calling you by name, they call you "case"; "this case here, case"...That is not a proper way. Why do you call me case? Am a being and I have a name. Am not a case and I have a right to be called my name. *(SSI, Key informant mental health service consumer)

*"...You find that stigma not only hinders seeking help, but it really tortures the patient a lot. It is actually more disabling than the illness itself *(SSI, Staff, National Mental Health User Association)

Poverty as a source of stigma

In broad terms, most respondents interviewed affirmed a relationship between poverty and stigma among people with mental illness in Uganda. Poor people with mental illness are more prone to stigma and other unfavourable consequences of mental illness than their counterparts with higher socio-economic status. Participants argued that the mentally ill from a poor background are more ostracized and rejected than those from families of higher socio-economic status

"...*Those ones with money are not affected. They can buy medicine, can rent, can do whatever they want. How will the mental illness affect them? They can get whatever they want. They are not stigmatized like us*" (SSI, mental health service consumer 4)

The participants further argued that in most societies, the poor are usually marginalized, irrespective of their mental status. One participant in the rural district was quoted as saying:

"...*what I know, if you are mentally ill, and you are from a good family, they can take care of you...maybe take you abroad or whatever...But if you are from a poor family, hah! You just wait until you die....(laughing)...maybe the vehicles knock you or what....but if you are from a good family, maybe they can take you to Butabika [psychiatric hospital] or where. Because even Butabika...somebody has to take you there. If you are rich, you get care, but if you are poor....hah!, you can't access any help... there it is really terrible. So you remain mad because of poverty" (SSI, Law and Justice Sector).*

Thus, in the opinion of some stakeholders, material resources can offer protection against stigma.

### Stigma, mental illness and the path to poverty

People with mental health problems who were interviewed confirmed that they were alienated by their family members and significant others due to their illness. They reported that after an episode of mental illness, relatives and significant others distance themselves and the consumers become socially excluded. Their social relationships thus tend to deteriorate very fast, leading to limited access to opportunities such as employment or other avenues of income generation. They further reported being excluded from a number of activities and being denied employment or other opportunities that could serve to enhance their economic well being:

"...*of course nobody can employ you if they know that you have mental illness. But if you get someone who doesn't know that you are a person with mental illness, he may employ you, and you will do his work well. But the moment someone tips him off that you have mental illness, I tell you, you will not last there. He will look for any excuse and eventually fire you*" (SSI, mental health service consumer 4).

Denying people with mental illness employment was reported to stifle the sufferers' chances of progressing financially and developing their careers. Some of the consumers admitted having lost jobs on being diagnosed with mental illness because of the associated stigma, leading to a dwindling socio-economic status. One user recounted:

"...*now like in public service...they used to have a question "Have you ever suffered from mental illness?"....it would have been a good question if they are going to help you on the job so that they will not overburden you. But it was a bad question used negatively because they would never call you for interview however much capability you had. Once you declared that you have ever suffered from mental illness, automatically you would be disqualified *(SSI, mental health service consumer 5).

According to some respondents people with mental illness are also less likely to send their children to school. Some participants cited earlier studies where this had been one of the major findings:

"...*again in that same study, we found that in a household, if someone (maybe the head of the family) had mental distress, the children were less likely to go to school. So, that makes that...the link is associational. But also if the children are not going to school, obviously the human capital of those children is going to be much less than it is in a family where there is no mental disorder. Because it is usually through education that people can improve their socio-economic status*" (SSI, Mental health professional, Academic and Researcher).

Similarly, according to the teachers who were interviewed, children with mental illness are less likely to attend and continue with school. If they are already in school at the time of onset of the mental illness, chances are high that they will be forced to drop out. This was attribute to two reasons. Firstly, such children often fall victims of stigma by schoolmates and teachers, prompting them to abandon school. Secondly, the parents might not only look at them as a disgrace but have very little hope in them and believe it is a waste of money to keep them in school. The resulting lack of education was believed to perpetuate trans-generational poverty.

The stigma manifested in the tendency to believe that mental illness is a permanent condition and those with the condition can never recover makes it hard for them to access financial services such as loans from micro-finance institutions. A number of participants noted that although it may not be a formally documented policy, micro-finance institutions do not extend loan services to people known to have a mental illness, thereby denying them opportunities to engage in income generating activities.

### Poverty, stigma and service utilization

According to many respondents, poverty also dictates the extent of mental health service utilisation. Access to better mental health services was reported to be extremely hard for the poor, especially those in the remote areas. Furthermore, even where free services are available in public health facilities, transport costs remain a major obstacle, particularly for those living in remote rural areas. This prolongs the period for which the poor people will battle with their mental illnesses, worsening the effects and making it more likely that their condition will become chronic, thereby exposing them to more stigma.

Stigma was reported to affect disclosure of the illness, which results in delayed help-seeking. Some users reported being aware that many people are uncomfortable identifying with mentally ill people. They maintained that this often prompts them to deny suffering from mental illness, and to decline seeking help at mental health facilities.

Some general health workers reported that some people with mental illness and the carers do not only avoid seeking help at mental health facilities but also conceal information about the mental illness:

"...*they prefer not to disclose or share details of their mental illness. As you take history, you may realize that it is mental illness. But when you ask, they deny. They deliberately decide to give confusing history of the problem*" (SSI, PHC doctor, urban district).

It was also pointed out by some respondents that consumers from higher socio-economic groups tend to seek as much privacy as possible, and make use of private sector facilities, where fewer people will therefore get to know about their condition. They do not want to identify with public mental health facilities because of the attached stigma. By seeking help from private facilities however, costs of care are increased. This high expenditure, coupled with the recurrent nature of mental illness, was reported to have negative financial consequences.

### Differing perspectives between stakeholders

It is important to note the varying perspectives that emerged between stakeholders regarding poverty, stigma and mental health. While some of the participants simply commented on stigma from the perspective of how they see people in the community treating those with mental illness, users were more likely to talk about stigma of mental illness given their unfortunate experiences of being victims of stigma. They commented on stigma in a rather disheartening manner, expressing views of stigma as a barrier to help seeking and service utilization. The policy makers on the other hand were more likely to suggest what could be appropriate strategies for averting stigma.

Participants from within the health sector mainly commented on stigma as a major hindrance to effective service delivery. Unlike other participants, they seemed to show more sympathy possibly due to a better appreciation of the experience of mental ill-health. On the other hand, some of the participants outside the health sector suggested strategies that would propagate stigma, as a means of ostensibly fighting stigma. This was a clear indication of low appreciation for mental health issues, which has been shown to be one of the challenges to mental health service delivery in low income countries. In one instance, a magistrate proposed keeping people with mental illness confined in institutions as one effective way of fighting stigma. The findings thus underscore the high investment and effort required to fight the deep rooted stigma reported by a range of stakeholders in Uganda.

## Discussion

According to most stakeholders interviewed in this study, there is a strong relationship between poverty and mental illness; the two being mostly linked in a vicious cycle of exclusion, limited access to services, low productivity, asset depletion and diminished livelihood. This is in line with a number of earlier studies that describe the relationship between poverty and mental ill-health [5-7,21,23-25].

The findings further revealed that social stigma is a powerful mediating factor in the relationship between poverty and mental illness. Nearly all participants commented on stigmatization as a pervasive reality in the lives of mentally ill people. Although some did not comment about stigma in detail, it was clear that the most participants believed mental illness is the most stigmatized of all disabilities. It was also noted that users tend to have preconceived feelings of rejection even before they are subjected to stigmatizing tendencies. The stigma attached to mental illness in this study is consistent with that documented in numerous studies and reports world wide [1-3,26,27]. A local survey by Basic Needs, Uganda (2005) revealed that many families do not want to be identified with the mentally ill relatives and that when patient does not show improvement fast, he/she is abandoned. The belief that mental illness is incurable and mostly caused by bad spirits exacerbates and intensifies the stigma and exclusion.

According to the study findings, the social relationships of people with mental illness deteriorate very rapidly as family members and friends often dissociate themselves from the person with mental illness due to stigma, thus leading to a spiral of reduced social support and increased social isolation. In conditions of poverty, these social relationships are affected both through direct material constraints and the shame associated with poverty. Earlier studies have affirmed a correlation between social support and income in the general population [25]. Since mental illness impairs users' interpersonal functioning, cutting down the users' social networks [28], the link between social stigma and poverty becomes more obvious. This was clearly articulated by many of the stakeholders in this study, especially the users.

An important finding was that users and other participants reaffirmed denial of access to credit services for people with mental illness. This is in line with earlier findings by Ntale [15], that while there is no evidence of an official policy by financial institutions to exclude disabled people from accessing loans, most disabled people are denied credit facilities in nearly all financial institutions. These findings are in line with comments by Saraceno & Barbui [5], who argue that poverty acts through economic stressors such as unemployment and lack of affordable housing, preceding mental illnesses such as depression and anxiety, making poverty an important risk factor for mental illness as well as a consequence of mental illness.

The interviews generated two insights concerning the reasons for denying people with mental illness access to credit services. First, these people are believed to have impaired functioning and to be unable to meaningfully engage in productive work. They are thus believed not to be in a position to pay back loans. Secondly, individuals with mental illness would not be charged before the law in case they defaulted. Therefore financing institutions would prefer not to risk their money with people who could easily be acquitted for reasons of insanity, thereby causing financial losses. This practice has important implications for people with mental illness: access to micro-credit services is very necessary, especially for the rural poor who need the support as they attempt to escape poverty through income generating activities. Furthermore, decreased access to social and financial services has been found to increase the disability [4].

Despite the fact that mental health consumers are disproportionately impacted by poverty, income and material resources are often not given prominence in mental health policy [8,29,30]. There is currently no disability grant for people with mental disorders in Uganda, an issue which requires urgent attention in mental health policy. While the challenge of serious mental illness should not be downplayed, broader recognition of the role of material resources in consumers' lives is needed [11]. A failure to recognize poverty as a key mental health policy issue will continue to constrain efforts to facilitate consumer empowerment and social integration [29].

It was noted that poverty impacts on help seeking behaviors as patients may not be able to finance themselves to the health facilities especially if they are deep in the villages. This observation is in line with many other studies that have confirmed this link. Fauerstein [25] argued that the poor often live far from where care is offered and that the poorer the mentally disordered, the greater the burden. Some participants particularly from the rural district affirmed that people find it hard to spend their little money seeking medical services before the condition has worsened. In line with this, Fauerstein [25] argues that the poor are usually reluctant to seek medical help and value their physical health more than their mental health. The study findings thus seemed to suggest a vicious cycle of mental illness, stigma and poverty.

## Limitation of the study

One major limitation of this study, is the fact that family members of people with mental illness were not interviewed as a separate group of stakeholders, although several of the stakeholders interviewed were also family members of service users. Family members should be included in future in such research, as a key stakeholder group.

## Conclusion

Mental health stakeholders in Uganda report that a strong interrelationship exists between poverty and mental illness, with stigma playing a crucial mediating role. The users reported on poverty and stigma issues more than any other group of participants, as they have more direct and personal experience than most other stakeholders. Poverty, stigma and mental illness therefore seem to inter-relate in a vicious cycle, to the disadvantage of poor people with mental illness. These findings thus re-affirm the need to recognize material resources as a central element in the fight against stigma of mental illness, and the importance of education and stigma reduction programmes in protecting those with mental illness from social isolation, particularly in conditions of poverty.

## Competing interests

The authors declare that they have no competing interests.

## Authors' contributions

SJ is the Research Officer on the project. He collected and analyzed data, with input from other colleagues working on the project. He drafted the paper and is the lead author. FK is the Principal Investigator. He participated in the design of the study, and data analysis. He has been actively involved in the editing of this paper. CL is the project coordinator. He participated in the design of the study, data analysis, search for literature, as well as editing of the paper. DK is a Research Assistant. She participated in the data collection and analysis, as well as editing and alignment of the paper. EO conceived the paper. She participated in the literature search for background information, sequence and alignment of the paper. The listed MHaPP group members conceived this study and were jointly involved in designing the data collection and analysis methods.

## Authors' information

JS is a Clinical Psychologist and is currently a Research Officer on the Mental Health and Poverty Project. He is also a part-time Assistant Lecturer in the Department of Mental Health and Community Psychology, Makerere University.

FK is a Senior Consultant Psychiatrist and Executive Director of Butabika National Referral Mental Hospital, Uganda. He is the Ugandan Principal Investigator on the Mental Health and Poverty Project. He is also the President Uganda Psychiatric Association, and formerly WPA zone 14 (East and Southern Africa) Representative.

CL is a Clinical Psychologist and Chief Research Officer in the Mental Health and Poverty Project, Department of Psychiatry and Mental Health, University of Cape Town, South Africa. He holds a PhD from the same University.

DK is a Senior Clinical Psychologist at Butabika National Referral Mental Hospital, Uganda. She is currently pursuing Doctoral studies at Norwegian University of Science and Technology.

EO is a Social Anthropologist and Senior Lecturer in the Department of Psychiatry, Makerere University Medical School, Uganda. She holds a PhD from Karolinska Institute, Sweden.

## Pre-publication history

The pre-publication history for this paper can be accessed here:

http://www.biomedcentral.com/1472-698X/9/5/prepub
